# Could the New BA.2.75 Sub-Variant Cause the Emergence of a Global Epidemic of COVID-19? A Scoping Review

**DOI:** 10.2147/IDR.S387551

**Published:** 2022-10-31

**Authors:** Nour Shaheen, Abdelrahman Mohamed, Almoatazbellah Attalla, Rehab Adel Diab, Sarya Swed, Abdulqadir J Nashwan, Ala’ Abdala Rababah, Mahmoud Tarek Hefnawy, Youssef Soliman, Omar Ahmed Abdelwahab, Mariam Tarek Desouki, Abdulrhman Khaity, Ahmed Shaheen, Abdelraouf Ramadan, Mostafa Meshref

**Affiliations:** 1 Alexandria Faculty of Medicine, Alexandria University, Alexandria, Egypt; 2 Faculty of Medicine, Al-Azhar University, Cairo, Egypt, Medical Research Group of Egypt, Cairo, Egypt; 3 Aleppo University, Faculty of Medicine, Aleppo, Syria; 4 Hamad Medical Corporation, Doha, Qatar; 5 Internal Medicine, King Hussein Medical Center, Amman, Jordan; 6 Faculty of Medicine, Zagazig University, Egypt, Medical Research Group of Egypt, Cairo, Egypt; 7 Faculty of Medicine, Assiut University, Assiut, Egypt; 8 Faculty of Medicine, Elrazi University, Khartoum, Sudan; 9 Faculty of Medicine, Helwan University, Cairo, Egypt; 10 Neurology Department, Faculty of Medicine, Al-Azhar University, Cairo, Egypt

**Keywords:** outbreak, Omicron, subvariant, BA.2.75, COVID-19

## Abstract

With over 58 million cases and 6 million deaths by August 2022, the Coronavirus disease 2019 (COVID-19), causing severe acute respiratory syndrome coronavirus 2 (SARs-CoV-2), has had an insurmountable impact on the world’s population. This is one of the worst health crises since 1918’s influenza pandemic. There are four subvariants of Omicron; BA.1, BA.1.1, BA.2 and BA.3. As a result of new mutations in its spike protein, most of which occur in its receptor binding site, the Omicron variant appears to be more transmissible and less resistant to vaccination and antibody response. Understanding Omicron’s virology and mutations is essential to developing diagnostic and therapeutic methods. A thorough assessment of control measures, as well as timely adjustment of control measures, requires addressing such issues as re-infection risk, vaccine response, booster vaccine doses, and the increased rate of Omicron infections. This review article aims to look at the current information about the different types of SARs-CoV-2, focusing on the new subtype BA.2.75.

## Introduction

SARS-CoV-2, which first emerged in the Chinese city of Wuhan, is infectious in humans and has spread quickly through intimate contact with other people or the release of respiratory secretions (ie, coughing or sneezing) from infected individuals. On March 12, 2020, the World Health Organization (WHO) Director-General proclaimed the COVID-19 outbreak “a pandemic” due to the rising infection rate in China.^1^,^2^ According to statistics, the enormous SARs-CoV-2 genome will produce single-point mutations daily due to the pandemic’s scope.^3^ Thousands of distinct mutations have been identified, and roughly a year after the outbreak began, strains with several mutations, mainly in the spike (S) gene, started to appear.^4^ These are the main variants of concern (VOCs).^5^ As a result, multiple waves occurred: The first wave (Alpha), the second wave (Delta), and the third wave (Omicron). Although Beta and Gamma caused regional outbreaks in southern Africa and South America, they did not spread globally.^6^ In November 2021, the Omicron variant was discovered in Botswana. It was reported from South Africa on November 24, 2021, and on November 26, 2021, it was classified as a VOC.^7^

Since the beginning of SARS-CoV-2, more than 2.2 million virus sequences have been generated and shared in online databases like GISAID. These genome sequences enable researchers to track, analyze and detect possible mutational variants.^8^

In addition to other proteins like NSP12 and NSP14 that are necessary for viral replication, Omicron contains a significant number of mutations that have previously been reported in other VOCs, including at least 32 mutations in the spike protein alone in contrast to the 16 mutations in the delta variant, which was already extremely contagious. The Omicron variant is more transmissible than other variants and the original SARS-COV-2. After that, Omicron sub-variants were identified in sequence. BA.1 was first discovered, followed by BA.2 sub-variant, then BA.4, and finally BA.5.^9^ Close epidemiological monitoring of newly emerging variants and lineages is also advised in the event of an increase in severe disease outcomes, such as an increase in hospitalization or ICU admissions. Evaluation of the efficacy of vaccines and antibody-based therapies against these new variants is also required. Also, the design and development of next-generation vaccines which are more effective and newer monoclonal antibodies to halt the spread of the continuously evolving and emerging newer SARS-CoV-2 variants and lineages.^10^

An increasing prevalence of BA.2.75 Omicron sub-variant in India and other countries like the UK, Canada, the US, Australia, and Japan might be behind the current COVID-19 surge. The (WHO) and experts have emphasized that countries should raise concerns about this new sub-variant because it has many spike mutations, manifests rapid growth, and has an extensive geographical distribution. In addition, BA.2.75 may have a crucial role in evading vaccine-induced immunity.^11–14^

Each Omicron sub-variant has specific characteristics. This study aims to describe each sub-variant in terms of data and country of discovery, transmissibility, spike mutations of interest, hospitalization rate, effect of vaccination, most significant symptoms reported, and diagnosis. In addition, we will focus on the new BA.2.75 sub-variant.

### Epidemiology

#### Covid-19 so Far

**Figure 1:**
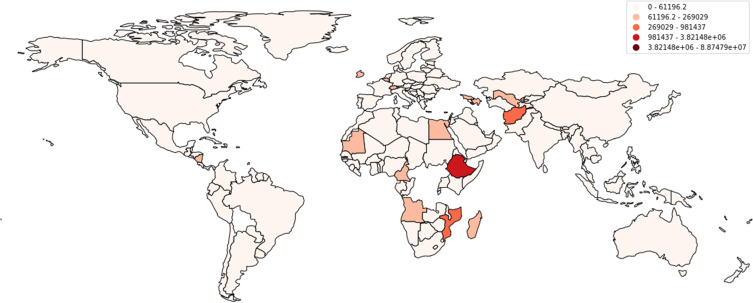
Is the cumulative number of deaths caused by COVID-19 as reported by WHO globally.

**Figure 2:**
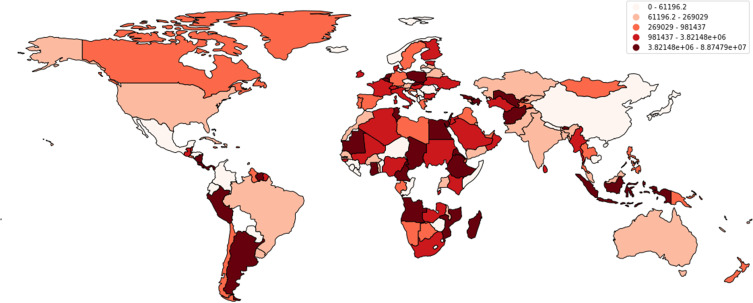
Shows the cumulative number of COVID-19 cases reported around the globe.

**Figure 3:**
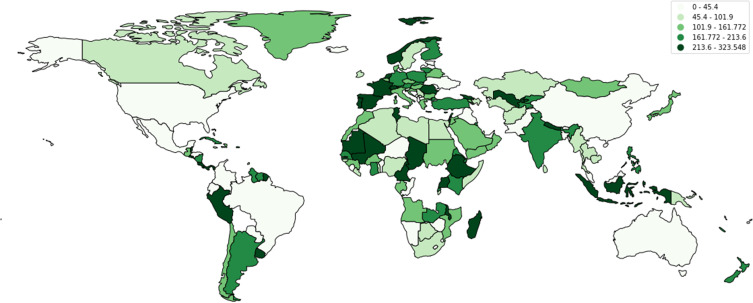
The total number of vaccinations per 100 people in the world.

**Figure 4:**
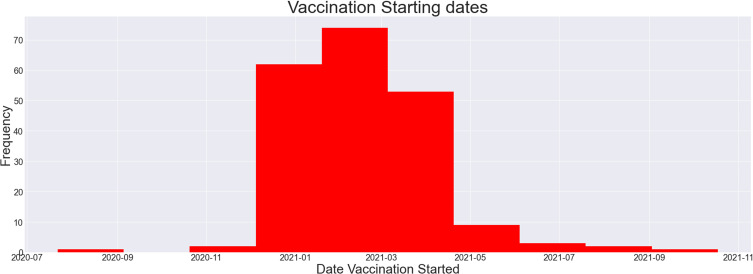
Distribution of vaccination starting dates in countries around the world.

According to data reported by the WHO, a cumulative total of 88,747,875 cases have been reported in the USA alone, with a cumulative total of 1,015,093 deaths in the past years (Figures 1–3). Multiple measures have been monitored by the WHO, including mask policies, travelling restrictions, social distancing, and, most importantly, vaccinations. Vaccinations started in July 2020 in China and have been made available worldwide since then (Figure 4).^2^,^15^

### Omicron Sub-Variants

**Figure 5:**
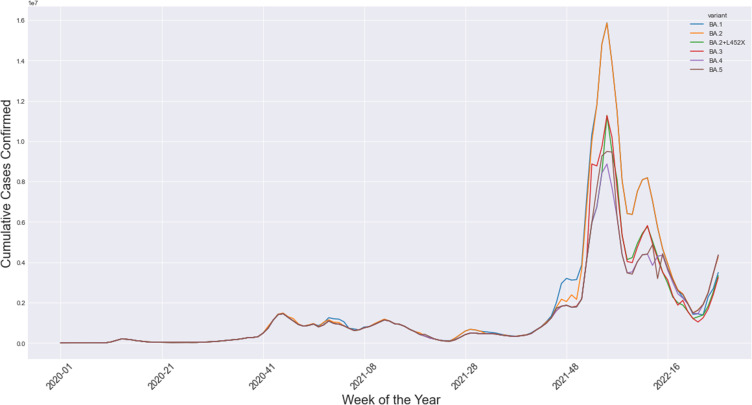
The cumulative number of cases confirmed in Europe as reported by ECDC from January 2020 up to July 2022.

**Figure 6:**
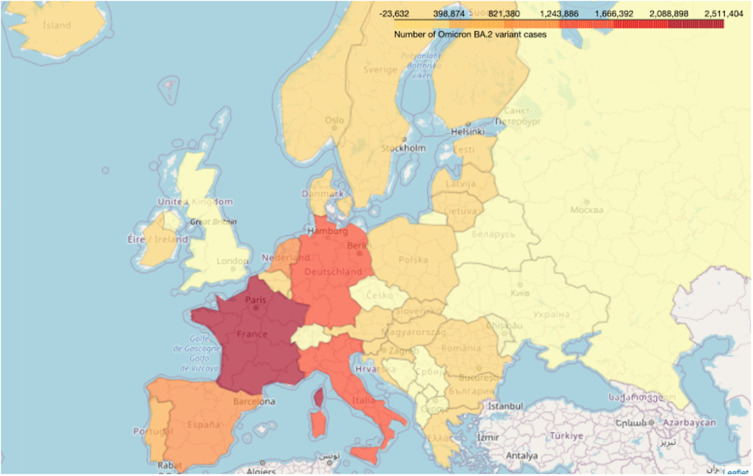
Map showing the sum of new BA 2 cases reported per week in different European countries from Jan. 2020 to July 2022.

**Figure 7:**
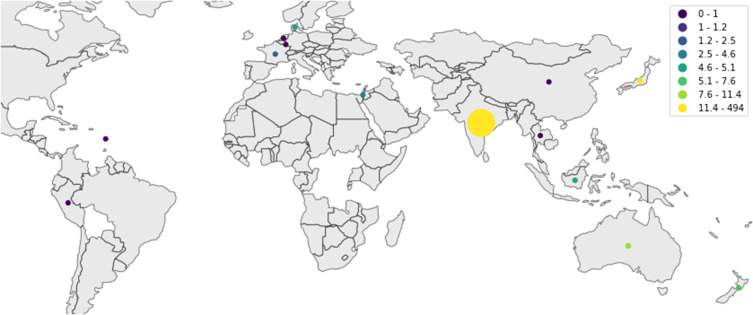
A map of the countries where BA.275 has appeared in the last few weeks.

**Figure 8:**
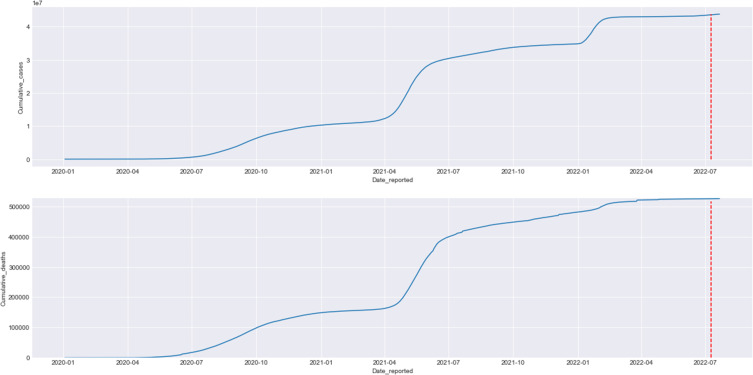
Visualizes the trend of COVID-19 cumulative cases and deaths in India from January 2020 to July 2022. The red dotted line marks.

Omicron’s four sub-lineages are considered variants of concern: BA.1, BA.2, BA.4, and BA.5. BA.2.75 is the new emerging variant. In the United States, the dominant omicron sub-variant is BA.2.12.1; in South Africa, the dominant is BA.4 and BA. 5.^8^ The Omicron sub-lineages (BA.1, BA.2, and BA.3) nearly appeared at the same time and location. However, BA.1 was dominant in infectivity and spread, ousting the Delta variant in late 2021. The BA.2 variant replaced it in March 2022 and peaked in April 2022. BA.3 was the least lineage in both infectivity and spread due to having no unique mutations in its spike proteins compared with BA.1 and BA.2 lineages^16^,^17^ (Figures 5–8).

### Immune Reaction of Omicron Sub-Variant

The Omicron variant is distinguished by 26–32 spike protein mutations, many of which are found within the receptor binding region. In addition, Omicron possesses mutations beyond the spike protein, three deletions, and one insertion in the spike protein.^18^ Work on previous VOC has shown that such variants may be substantially different antigenically, and many of the changes are either known or projected to contribute to the escape from neutralizing antibodies.^19^ A preprint paper found increased immune escape of the Omicron variant.^20^

The risk of reinfection by the Omicron sub-variants is considered a question of great importance. It depends on the ability of the new sub-variants to escape the immune system. According to laboratory research, the convalescent serum seems to have a diminished neutralizing effect on the Beta and Delta variants of the virus in vitro compared to the wild-type virus.^18^ However, this does not necessarily translate into immunological escape at the population level.^19^,^21^,^22^

### Clinical Presentation and Severity

The severity of the Omicron variant seems to be less than the delta variant, even though they have similar clinical manifestations.^23^ According to preliminary evidence, Omicron may produce relatively mild sickness, with some patients developing severe illness, requiring hospitalization, and dying due to the infection caused by this variant.^7^,^23^ The hospitalization rate increased with all Omicron sub-variants.^24^ Although there was little increase in hospitalization and death rates in South Africa, outside of it, there was a significant increase in both.^25^ The most frequent complaints of patients are fever, cough, exhaustion, and loss of taste and/or smell. Other symptoms include pharyngitis, headaches, skin rashes, discolored fingers and toes, red or itchy eyes, stomach cramps, and diarrhea are frequently seen with Omicron. It may also result in serious symptoms like dizziness, shortness of breath, chest discomfort, difficulty moving, and difficulty breathing. Respiratory symptoms include cough, scratchy throat, shortness of breath, and pneumonia may also be seen.^26^

### Country and Date of Detection

#### BA.2.75

The new omicron sub-variant BA.2.75 was first detected in early June in India and identified as a variant of concern by the WHO on July 7, 2022,^27^ At least 23 samples of the BA.2.75 variant in India have been recorded in Karnataka, Maharashtra, and Jammu & Kashmir. About 37 samples have been detected in Australia, Canada, Germany, and New Zealand.^28^ There are two cases of BA.2.75 in the United States, the first of which was identified on June 14, but both have not been officially confirmed.^29^ It has then spread to more than ten countries, including the UK and Japan.^30^

### BA.1

According to the Phylogenetic tree analysis, Omicron independently evolved from the previous SARS Cov-2 variants as Alpha, Beta, Gamma, and Delta.^31^ Although the Omicron variant was initially reported in November 2021 in South Africa, the first confirmed case was in the Netherlands.^32^,^33^ BA1.1 emerged in late 2021 and rapidly became predominant in early 2022.^34^

### BA.2

BA.2 has been detected in at least 67 countries, and the most dominant variant is in the Philippines, India, and Denmark.^35^

BA.4 and BA.5 variants accounted for more than 50% of sequenced cases in South Africa^36^ (Table 1).

**Table 1:** Summarize the Characteristics of Each Omicron Sub-Variant

**WHO Label**	Lineage	Country First Detected	Date of Variant Discovery.	Transmissibility	Spike Protein of Mutations of Interest	Rates of Hospitalizations	Effect of Vaccination Booster Dose: Degree of Effectiveness in Terms of the Amount of Produced Neutralizing Antibodies	Most Significant Symptoms Were Reported.
**Omicron**	BA.1	Bothnia, South Africa	November 2021.^9^	Increased	A67V, Δ69-70, T95I, G142D, Δ143-145, N211I, Δ212, ins215EPE, G339D, S371L, S373P, S375F, K417N, N440K, G446S, S477N, T478K, E484A, Q493R, G496S, Q498R, N501Y, Y505H, T547K, D614G, H655Y, N679K, P681H, N764K, D796Y, N856K, Q954H, N969K, L981F.^9^	Increased^24^	A moderate degree of effectiveness. But still, taking booster doses is recommended by WHO to minimize mortality and hospitalization rates.^74^	Mostly cold- and flu-like symptoms. No significant symptoms were reported as it was more likely to be asymptomatic^50^
**Omicron**	BA.2	South Africa	November 2021.^9^	Increased 30–50% more transmissible than BA.1.^75^ But it was proved that BA.2.75 sub-lineage transmissibility is much high, competing with that of BA.5^76^	G142D, N211I, Δ212, V213G, G339D, S371F, S373P, S375F, T376A, D405N, R408S, K417N, N440K, S477N, T478K, E484A, Q493R, Q498R, N501Y, Y505H, D614G, H655Y, N679K, P681H, N764K, D796Y, Q954H, N969K.^9^	Increased^24^	A moderate degree of effectiveness. But still, taking booster doses is recommended by WHO to minimize mortality and hospitalization rates.^74^	Cough, fatigue, congestion, and runny nose^51^
**Omicron**	BA.4	South Africa	January 2022.^9^	Increased by 36% compared to the BA.2 lineage.^17^	L452R, F486V, R493Q.^9^	N/A (Mohapatra et al, 2022c) Although a small rise in hospitalization and mortality rates was seen in South Africa, outside it, a more rise in both mortality and hospitalization rates was witnessed.^25^	A mild degree of effectiveness, as there was seven folds drop in the neutralizing antibodies produced against both BA.4 and BA.5 compared to BA.1 But still, taking booster doses is recommended by WHO to minimize mortality and hospitalization rates.^17^	Very severe throat pain and increasing fatigue.^53^
**Omicron**	BA.5	South Africa	February 2022.^9^	Increased by 36% compared to the BA.2 lineage.^17^	L452R, F486V, R493Q.^9^	N/A.^17^ A small rise in hospitalization and mortality rates was seen in South Africa; outside it, a more rise in mortality and hospitalization rates was witnessed.^25^	A mild degree of effectiveness. As there was seven folds drop in the neutralizing antibodies produced against both BA.4 and BA.5 compared to BA.1 But still, taking booster doses is recommended by WHO to minimize mortality and hospitalization rates.^17^	Very severe throat pain and increasing fatigue.^53^

### Mutations of Interest and Sequencing of Omicron Sub-Variants

#### BA.2.75

The scientific director of WHO, Soumya Swaminathan, published a video about this sub-variant saying, “There are still limited sequences available to analyze, but this sub-variant seems to have a few mutations on the receptor binding domain of the spike protein”.^37^

This sub-variant shows a high number of mutations in genome sequences, mainly in the spike proteins of the virus with a high weakly growth with possible mutations at S: K147E, S: F157L, S: W152R, S: G257S, S.I210V, S: D339H, S: N460K, S: G446S and S: R493Q. The most concerning are G446S and R493Q. The G446S mutation can potentially affect immune evasion and ACE2 binding. Given the available evidence, it is possible that, although this mutation may reduce the binding effect of the sub-variant, it may also lead to considerable immunological escape. This suggests that reinfections and breakthrough infections may be driving the spread of BA.2.75. This may explain the increasing number of cases in India.^38^,^39^ BA.2.75 has been dominant in many regions worldwide lately, acquiring more rise in its alpha helices’ number than BA.2, which enables it to escape immunity.^40^,^41^

Another hypothesis is that a unique characteristic is acquired from APOBEC-induced mutations, which lead to the loss of 2 CPG dinucleotides, and the gain of 4 new ones in the Omicron BA.2 variant, thus escaping the zinc finger antiviral protein in lung cells.^42^ Another finding is that there is a high expression of transmembrane serine protease 2, which is generally involved in regulating cellular signaling in the plasma membrane and extracellular matrix. These molecules were found to be dominant in various cancers.^43^

According to the available online data in genome trackers platforms, the number of mutations in the BA.2.75 spike protein is more than those in the BA.4/5, making it highly contagious and resistant to neutralization compared with other omicron variants.^27^,^29^ However, researchers are still tracking its possible mutations.

### BA.1

**Figure 9:**
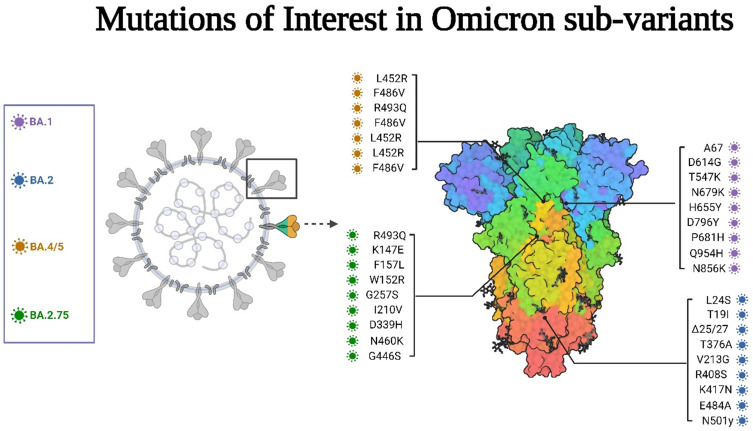
The SARS-CoV-2 Variants of Concern.

The classical Omicron variant (B.1.1.529) constitutes 18,261 mutations in its genome (Figure 9), most of them in the coding region, 97% of the total mutations, and most of these mutational events, 60%, are skewed towards the spike proteins as A67, D614G, T547K, N679K, H655Y, D796Y, P681H, Q954H, and N856K and others.^31^,^33^ In addition, BA.1.1 is different from BA.1 in the presence of a single mutation (R346K).^27^

### BA.2

BA.2 sub-lineage genome consists of substitutions and deletions in the Receptor binding site, S2 region, and furin cleavage domain.^35^ It is characterized by the key S mutations L24S, T19I, Δ25/27, T376A, V213G, and R408S.^44^ In addition, BA 2 variant exhibits 15 mutations in the receptor binding domain, which includes the mutations in K417N, E484A, and N501y, facilitating its escape from neutralizing antibodies, enhanced by the formation of new salt bridges and hydrogen bonds produced by R49, R498, which are mutated residues in the receptor binding protein with ACE2. This change was triggered by increased alpha helices in the RBP, increasing its susceptibility to several mutations.^43^

### BA.4 and BA.5

Spike mutations of interest are L452R, F486V, and R493Q. The S-proteins of the Omicron variants BA.4 and BA.5 are identical to the variant BA.2 except for 69–70 deletion, F486V, and L452R.^17^,^36^ BA.4 and BA.5 each have unique alterations, including L452R and F486V in the viral spike protein, which may affect the virus’s capacity to attach to host cells and evade various immune responses.^45^ As a result of the high similarity between BA.2 and BA4/5, it was found that many genome sequences classified as BA.2 in public databases are BA.4 or BA.5, thus underestimating the variants’ ongoing global rise^25^ (Table 1).

### Transmissibility of the Omicron Sub-Variants (BA.2.75)

The mechanism of transmission of the BA.2.75 sub-variant is still unclear, and there is no evidence of any difference between it and the BA.2 sub-variant. However, the current reports may support the hypothesis that BA.2.75 spreads faster, as the curve of new cases in India has increased since the identification of the BA.2.75 sub-variant.^46^ The daily cases in India have been less than 3000 for several months, but recently they began to increase and reached 18,000/day, which is 1/100K of the population^38^ (Table 1).

### Clinical Characteristics and Severity of the Omicron Sub-Variants

It is early to say anything about the severity of the new sub-variant, but it seems to be as severe as the BA.2 variant with a higher transmissibility rate. Further investigations are crucially needed. The first cases of the BA.2.75 sub-variant were asymptomatic, and all recovered at home.^28^,^47–49^ Further studies are required to investigate the clinical picture and severity of the new sub-variant and compare it with previous variants (Table 1).

As a result of the insufficient data reported on the new B.A.2.75 Omicron sub-variant, the evidence of its infectivity, immunity evasion, and severity is still unclear. However, investigations are proceeding to understand the details regarding the BA.2.75 sub-variant to suggest the best effective methods to restrict the upcoming surge.^11^,^49^

BA.1 seems to cause a mild form of the disease. The most significant symptoms reported with BA.1 sub-variant are cold and flu-like symptoms with no significant symptoms reported. Infections were more likely to be asymptomatic.^50^

BA.2 also causes mild illness but is more severe than BA.1. Symptoms with BA.2 subvariant are cough, fatigue, congestion, and runny nose.^51^ In addition, the impact of BA.2 sub-variant on immunity increased.^9^ A study by Chen et al 2022. Showed a very low prevalence of neutralizing antibodies against BA.2 sub-variant among the Hong Kong population.^52^

BA.4 and BA.5 cause more severe disease than BA.1 and BA.2.^24^ The most reported symptoms with BA.4 and BA.5 sub-variants were severe throat pain and increasing fatigue.^53^

### Clinical Diagnosis

The diagnosis of Omicron cases depends mainly on molecular and immunological testing. The molecular testing includes real-time PCR (RT-qPCR), Next generation sequencing, and rapid molecular detection by LAMP. RT-qPCR can identify Omicron but cannot differentiate between its sub-variants. It identifies Omicron by detecting mutations in the S gene. The focus of the RT-PCR diagnostic kits, which have been approved, is the E, Rd, Rp, and N genes^54^ (Bazargan et al).

RT-PCR gives positive or negative results. Therefore, positive results may be recommended to undergo further DNA sequencing to identify the new mutation, which leads to variant identification.^26^

Immunological testing includes testing for antigens or antibodies, but they are not confirmatory. Therefore, the sample is preferably taken from the upper respiratory tract or saliva. Antibodies measured are directed against S or N protein, ie, virus-specific antibodies.^26^

So as a general approach to diagnosis, we depend on RT-qPCR, and according to the results, we classify patients into A) Suspected: absence of E484K and L452R mutations in the Spike protein.

B- Probable: detection of Spike protein substitutions K417N, S371L-S373P, and Q493R, or deletion at position 69/70; then to confirm the diagnosis, we perform whole genome sequencing (WGS)^55^

### Expected Response to BA.2.75

Previous recommendations to tackle the COVID‐19 pandemic must be sustained worldwide with recently improvised instructions. These include vaccinations for all individuals, social distancing, mask-wearing, and careful handwashing and quarantining of the BA.2.75 sub-variant positive patients in an alternative area. It is critical to highlight that genome sequencing of all samples could potentially impact the control of disease and evaluate the threat of new waves in the world.^11–13^ Further surveillance is needed to prevent more dissemination of the BA.2.75 sub-variant.

### Vaccination Against BA.2.75

Despite the spread of the new variant in India and other countries, it is early to predict if it is a dominant strain. Still, it shows a rapid growth rate of 16% per day other than BA.2 sub-variants found in most reported cases in India.^39^,^56^ Therefore, adding boosting protection against BA.2.75 to vaccines is important to avoid the spike of BA.2.75 and its spread.

### Immunity Against BA.4 / BA.5

The COVID-19 vaccines have a mild effect on it, as there were seven folds drop in the neutralizing antibodies produced against both BA.4 and BA.5, compared to BA.1. Booster doses are recommended by the WHO to minimize the mortality and hospitalization rates.^60^,^61^

### Omicron Variants and Susceptibility to Vaccine

The Existing vaccines targeting the first COVID variant detected in Wuhan, China, showed effectiveness in decreasing the severity of infection.^57^ However, its effect diminishes over time and appears to be less effective in the new variants of Omicron declared by the Vaccines and Related Biological Products Advisory Committee (VRBPAC) meeting at the Food and Drug Administration (FDA) in late June 2022.^58^ Therefore, all these issues encouraged the pharmaceutical companies to make Omicron-specific vaccines to face all sub-variants and their modifications. These vaccines will be administered as booster doses^59^,^60^ In addition, vaccines have produced neutralizing antibodies against different omicron variants.^61^

Omicron BA.1 sub-lineage is more reliable to neutralize antibodies generated from vaccine combination compared to other lineages such as BA.2.12.1 and BA.5, which are less susceptible to neutralization by 2.4 to 5.3 times.^61^ A study by Tan et al has shown that the BNT16b2 Vaccine reduced infection and hospitalization from omicron subvariants B.1.1529 and B1.617.2 in Singapore with estimated effectiveness of 36.8% and 65.3%, respectively, among children from 5 to 11.^62^

Currently, the most dominant strain in the US is BA.5, representing 65% of cases in the US from July 3–9. It is Hyper contiguous and five times more resistant to vaccines, especially mRNA vaccines, as Moderna and Pfizer.^63^ Despite the lower efficacy of vaccines, it still provides protection compared to their absence. Non-vaccinated individuals are five times more likely to get BA5 sub lineage infection and 7.5 times higher to become hospitalized. However, BA.5 is becoming more prevalent over time, and the incidence of BA.1 and BA.2 strains is decreasing.^63^ Moderna and Pfizer are now updating booster vaccines against BA4 and BA5, Moderna mRNA-123.214. A clinical trial showed that this bivalent vaccine would be effective against both BA4 and BA5, and it is expected to be administered by October.^58^,^59^ ECDC released a statement on Omicron sub-variants stating that ECDC has reclassified Omicron sub-lineages BA.4 and BA.5 from variants of interest to variants of concern. The currently observed growth advantage for BA.4 and BA.5 is likely due to their ability to evade immune protection induced by prior infection and/or vaccination, particularly if this has waned over time^9^(Table 1).

### Spread and Prevention

#### Expected Spread of Omicron

According to the CDC’s hospital forecast, daily COVID-19 hospital admissions are likely to increase by July 2022.^64^ As a result, immunized patients, whether through vaccines or natural immunity, are at risk.

BA.2.75 will likely become a trend in the upcoming weeks. However, it is too early to conclude that this lineage spreads faster or will become the dominant one in the future.

### Prevention of Omicron and Subvariants

Although Omicron is more transmissible than the previous variants, it spreads through the same mechanisms. This primarily happens when a person is exposed to the respiratory fluids carrying the virus, whether, through inhalation of respiratory droplets or their deposition on mucous membranes^50^ In addition, the CDC continues to track the spread of Omicron and its sub-variants and advises the public on prevention methods.^65^

The most effective way to stop the increasing number of cases is through vaccination. Vaccines have decreased hospitalization and mortality and are our first line of defense in this pandemic.^66^ Up-to-date vaccination is achieved when an individual has received every dose in a primary series and the recommended boosters whenever eligible. The age, type of vaccine received, and time since the last dose determine further vaccine recommendations for the individual.^67^,^68^ In addition, mRNA-based vaccines have been shown to induce neutralizing immunity against Omicron and are highly recommended for their use as a booster dose.^69^,^70^

The CDC has also advised the public to wear a properly fitting mask to protect against the spread of COVID-19, including Omicron and other variants. The mask does not need to be worn outdoors but should be used when sick or are around or caring for someone with COVID. The recommendations also depend on the COVID-19 community level. The CDC has divided it into low, medium, and high. In low levels, a mask is worn based on personal preference and risk level. For those in a medium community level, it is advised to wear one if there is a risk for severe illness, based on what doctors recommend, and when coming in contact with someone at risk for severe disease. If the risk is high, masks should be worn indoors in public, regardless of vaccination and risk of hospitalization.^71^

People with a high risk of severe illness from COVID-19 are advised to keep 6 feet from others outside or in public. Close contact with sick individuals should be avoided at home, especially when caring for a sick person. It is also crucial to avoid crowds and poorly ventilated areas while keeping homes well-ventilated by opening windows and doors. Handwashing with soap for a minimum of 20 seconds when in public spaces or after coughing or sneezing. A hand sanitizer with at least 60% alcohol concentration may be used as a substitute if soap and water are unavailable. Sneezes and coughs should be covered, and surfaces should be cleaned and disinfected frequently for maximal protection.^72^

Testing for COVID-19 is recommended before and after travel, when coming in contact with an infected person, or if there are suspected symptoms, including fever, cough, fatigue, sore throat, nasal congestion, headache, or loss of taste/or smell.^73^

## Conclusion

The SARS-CoV-2 virus, which continues to evolve, is unlikely to disappear completely in the near future. COVID-19 is predicted to eventually be dominated by the Omicron variant, which causes milder symptoms than seasonal influenza. COVID-19 has been expected to become endemic due to the appearance of the Omicron subvariants, which cause mild symptoms despite being more contagious. Vaccination has been shown to reduce the risk of long-term COVID-19 infection. Therefore, vaccination advocacy is still necessary in order to protect individuals who are particularly susceptible.

## Abbreviations

WHO, World Health Organization; VOC, variant of concern; VRBPAC, Vaccines and Related Biological Products Advisory Committee; FDA, the Food and Drug Administration.

## Data Sharing Statement

All data generated during this study are included in this published article.
